# A systematic review of oral wound healing indices

**DOI:** 10.1371/journal.pone.0290050

**Published:** 2024-02-08

**Authors:** Amanda Beatriz Rodriguez, Sara Alhachache, Diego Velasquez, Hsun-Liang Chan

**Affiliations:** 1 Department of Periodontics and Oral Medicine, School of Dentistry, University of Michigan, Ann Arbor, Michigan, United States of America; 2 Department of Periodontics, University of Louisville School of Dentistry, Louisville, Kentucky, United States of America; 3 Private Practice, Fenton, Michigan, United States of America; Justus Liebig University Giessen, GERMANY

## Abstract

Wound healing monitoring for abnormality identification and intervention is crucial to securing a successful surgical outcome. Indices have been used to summarize the degree of healing. Given the increasing frequency of regenerative procedures which preserve dentition and implant stability, and the higher esthetic demands, an appraisal of the available indices is needed to identify the current knowledge gap. This study aimed to systematically review published oral wound healing indices and scores. **Materials and Methods**: A complete literature electronic search in 5 databases was conducted by two reviewers. A combination of keywords related to oral wound healing was used. **Results:** A total of 11 articles were included in the evaluation of various procedures (conventional periodontal procedures, guided tissue regeneration, soft tissue reconstruction procedures, and tooth extractions), at different time points (1 day to 12 weeks), with a focus on diverse clinical signs and symptoms. Frequently evaluated parameters included wound dehiscence/epithelialization (91%), tissue color (redness) (73%), suppuration (55%), swelling/edema (55%), and hemostasis (55%). Other less commonly used parameters include esthetics-related and patient-centered outcomes. **Conclusion:** The available indices evaluate a diverse group of subjective clinical signs and symptoms to estimate the underlying biological healing events and assess the degree of clinical success. The majority of the included indices are not validated. Quantitative and objective subclinical parameters including blood perfusion, biomaterial stability, and completeness of epithelialization, are needed for customized wound healing care and better outcome prediction.

## Introduction

Surgical healing is a complex process of repair or regeneration of treated tissue which occurs in four main phases: hemostasis, inflammation, proliferation, and remodeling [1]. Hemostasis occurs immediately upon injury, starting with vasoconstriction and platelet activation [2,3]. Inflammation occurs soon afterward and entails the removal of necrotic tissues to prevent and fight bacterial invasion. The fibrin clot also serves as a scaffold for cell migration and population. During the proliferative phase, there is new tissue growth. The epithelium is among the first tissue to react, typically 2–4 days post-surgery. Additionally, fibroblasts lay down collagen matrices to fill in the wound edge and the excised tissue void [3,4]. Remodeling is the final phase and can last months. During remodeling, there is continuous collagen turnover to build the tensile strength and elasticity of the new tissue [2,5]. When hard tissue is involved, it will undergo catabolic and anabolic phases for the bone tissues to consolidate. These events concur with soft tissue healing [6].

Wound healing is crucial for successful periodontal and implant surgery, especially for soft and hard tissue regenerative procedures which are more commonly practiced nowadays [7]. Favorable wound healing starts with early formation, organization, stabilization, and attachment of the blood clot. It must be able to resist mechanical forces acting on the wound. The degree of clot stability is influenced by the host response, local anatomical features, constant bacterial challenge, surgical trauma severity, and wound closure management [7,8]. Sutures, used to close the wound edges, must remain stable and passive to ensure proper closure without impairing the physiological aspects of wound healing [9,10]. A harmonious transition between the healing phases and continuous wound stability ensures that wound healing is advancing properly. Prolonged healing or deviated healing events may result in increased complications, patient morbidity, and procedure failures, which negatively affect patients’ quality of life. Therefore, wound healing monitoring for early identification of abnormalities and interventions is a necessity [7,11].

Clinically, wound healing is assessed by examination of the surgical site and understanding the patient’s symptoms. Wound healing indices have been described which summarize and quantify the degree of healing from the evaluation of the selected clinical signs and symptoms [5,11–13]. The common end goals are the complete soft and hard tissue healing, absence of abnormal clinical symptoms, and free of postsurgical complications [7,11,14]. Given the increasing frequency of applying periodontal and implant-related regenerative procedures, improved understanding of wound healing, higher demands for esthetics from patients, and the recent focus on patient-centered reports, an appraisal of the currently available indices deems necessary. The present work aimed to systematically review published oral wound healing indices and scores related to periodontal and implant surgeries.

## Materials and methods

The Preferred Reporting Items for Systematic Reviews and Meta-Analyses (PRISMA) guidelines/checklist were followed during the present systematic review. An electronic search was conducted by two reviewers (A.R. and S.A.) on the following databases: NLM PubMed, Embase, EBSCOhost CINAHL, EBSCOhost Dentistry and Oral Sciences Source, and Wiley Cochrane Central Register of Controlled Trials. This search was performed from June 2021 to March 2023. The working PubMed search was as follows: (wound healing[mh] OR wound*[tiab]) AND (dental implants/surgery[mh] OR periodontal diseases/surgery[mh] OR ((gingiv*[ti] OR implant*[ti] OR periodontal[ti] OR soft tissue[ti]) AND (oral surgical procedures[mj] OR incision*[ti] OR invasive[ti] OR noninvasive[ti] OR operation[ti] OR operations[ti] OR operativ*[ti] OR procedure*[ti] OR surger*[tiab] OR surgic*[tiab]))) AND (monitoring, physiologic[mh] OR postoperative care[mh] OR applicat*[ti] OR assess*[ti] OR emdogain[tiab] OR examinat*[ti] OR index*[tiab] OR maint*[ti] OR monitor*[tiab] OR observat*[ti] OR predict*[tiab] OR progno*[ti] OR program*[ti] OR scale[tiab] OR scales[tiab] OR score*[tiab] OR system[ti] OR systems[ti]).

### Focused question

In periodontal, implant, and oral and maxillofacial surgical procedures, what are the indices, and scores used to monitor wound healing?

### Eligibility criteria

Studies were selected according to the following criteria:

### Inclusion criteria

- Human clinical studies that use or propose a specific index or score to evaluate wound healing of periodontal, implant, or oral and maxillofacial surgeries

### Exclusion criteria

- Studies using parameters not specific for assessing wound healing, such as probing depth, plaque index, etc.
- Review articles, including workshop consensus
- Articles not written in English

### Data extraction

Data were manually extracted by the same examiners (A.R. and S.A.), independently. In case of a disagreement, a third (D.V) and fourth examiner (H.L.C) reviewed the data in question and an agreement was reached by consensus. The methodological quality of the studies was assessed with the Cochrane Risk of Bias Tool. Two researchers independently evaluated the quality of the included studies (A.R., S.A.). A third and fourth researcher (D.V and H.L.C) were utilized in case of a disagreement.

## Results

### Study selection

**Fig 1:**
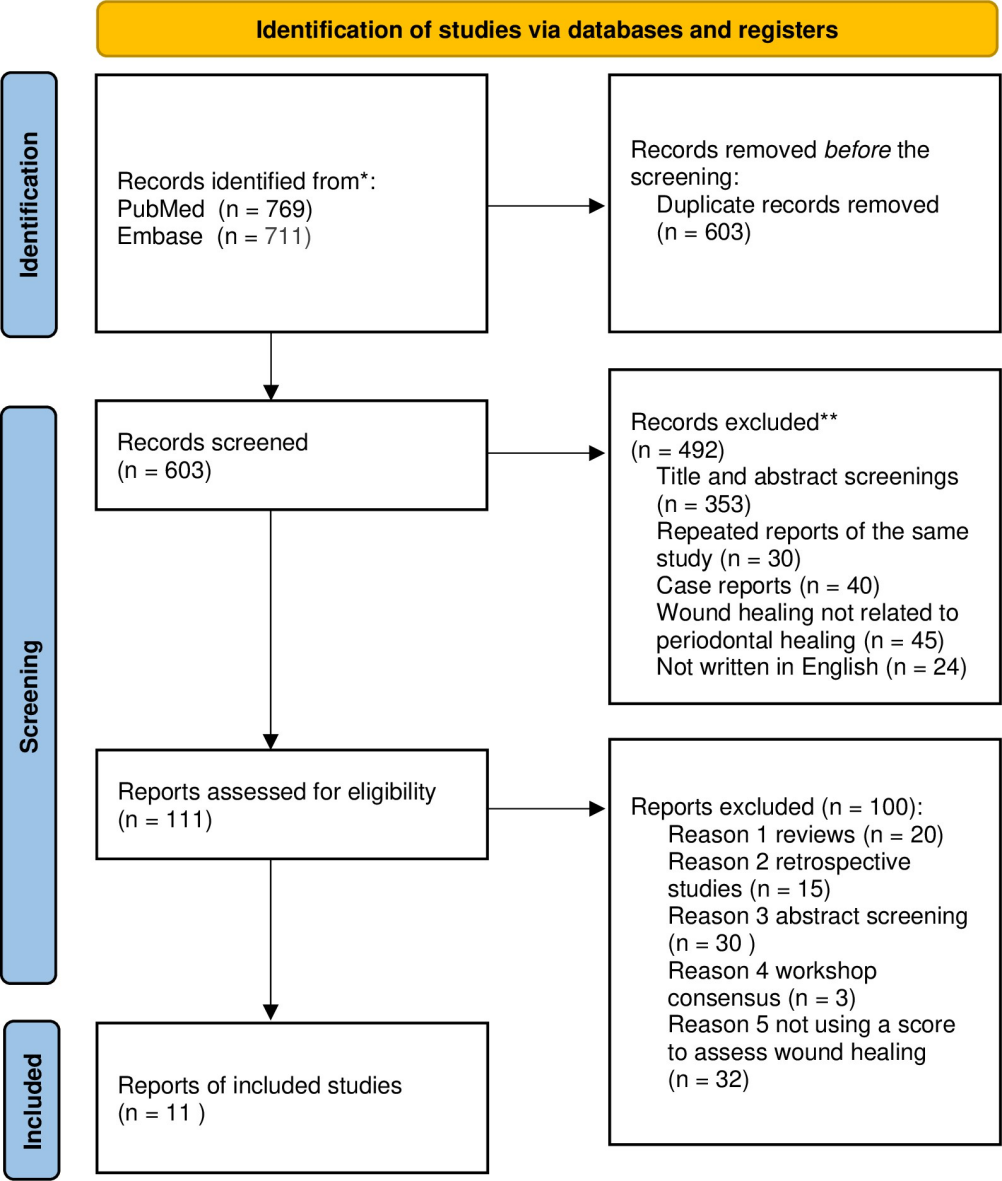
Search strategies for the systematic review.

The literature selection process is illustrated by a PRISMA flowchart (Fig 1). Initial screening yielded a total of 711 articles in PubMed and 769 in the EMBASE database search. References from these three searches were combined, and after removing the duplicates, 603 articles were available for title and abstract review. Of these, 492 articles did not meet the inclusion criteria and were excluded. Following a full-text review of the remaining 111 articles, another 100 articles were excluded. A total of 11 articles were included and summarized in Table 1. Cross-checking the reference list revealed that no articles were missed by the initial search. Considerable heterogeneity in the evaluated parameters was found, which precluded quantitative data analysis. The included indices are summarized in order of the publication time.

**Table 1:** Summary of wound healing indices included in the systematic review.

Author (year)	Patient numbers	Indication	Time evaluated	Wound healing index/score name	Parameters	Scores
Landry et al. 1988 [13]	N/A	Periodontal surgery	2nd and 4th weeks	Healing index (HI)	Tissue color, response to palpation, granulation tissue, suppuration, and incision margin	5 levels: Score 1, very poor (with presence of 2 or more signs) >50% of red gingiva, presence of granulation tissue, bleeding on palpation and suppuration, and incision margins not epithelialized, with loss of epithelium beyond incision margin. Score 2: poor healing with >50% of red gingiva, presence 2 signs, and incision margins not epithelialized, with exposed connective tissue. Score 3: Good healing with 25–50% red gingiva, absence of signs and incision margin does not expose connective tissue. Score 4: Very good healing with <25% of red gingiva, absence of signs and incision margin does not expose connective tissue Score 5: Excellent healing with all pink tissue color, absence of signs and incision margin does not expose connective tissue
Quinn et al. 1998 [16]	G1: 91 G2: 43	G1: Facial lacerations G2: Head/Neck Vertical releasing incisions	1 month	Wound evaluation scale (WES) and VAS	Step-off borders, contour irregularities, wound margin separation, edge inversion, inflammation\overall cosmetic appearance/scar visibility	WES: assessing 6 clinical variables (0 for yes and 1 for no) a total score of 6 was considered optimal, while a score of < 5 suboptimal) VAS cosmetic scale: (0-worst possible scar, 100- best possible scar)
Wachtel et al. 2003 [17]	11	Microsurgical access flap (GTR)	1 and 2 weeks	Early Wound Healing Index (EWHI)	Flap closure not only as complete or incomplete, amount of fibrin and necrosis	5 degrees: 1: complete flap closure–no fibrin line in the interproximal area 2: complete flap closure–fine fibrin line in the interproximal area 3: complete flap closure–fibrin clot in the interproximal area 4: incomplete flap closure–partial necrosis of the interproximal tissue 5: incomplete flap closure–complete necrosis of the interproximal tissue
Hagenaars et al. 2004 [12]	22	Periodontal flap surgery	Surgery day, 1, 4 and 8 weeks	VAS	Swelling of soft tissue, color of the gingiva, PPD, CAL, BI, PI	3-point score (both from 0 to 2) assessing swelling and color of the gingival tissues
Tonetti et al. 2004 [18]	172	Periodontal surgery	1, 2, 3, 4, 6 and 12 weeks Patient perception: surgery day, 1 week and 1 year	Composite index	Pain, wound closure, wound dehiscence, presence of granulation tissue at wound margin, suppuration, root sensitivity, edema, hematoma. Patient perception: chewing comfort, esthetic appearance, speaking ability oral hygiene ability, overall satisfaction	• Visual analogue scale (VAS) for the intensity of pain and discomfort: score of 50 = no change and a score> 50 indicating an improvement. • Presence or absence (dichotomous) of stringent parameters
Huang et al. 2005 [19]	23	Soft Tissue periodontal surgery	10 to 14 days and 1, 3, and 6 months after the surgery.	Wound Healing Index (WHI)	Gingival edema, erythema, suppuration, patient discomfort, and flap dehiscence	• Score 1 = uneventful healing with no gingival edema, erythema, suppuration, patient discomfort, or flap dehiscence. • Score 2 = uneventful healing with slight gingival edema, erythema, patient discomfort, or flap dehiscence, but no suppuration. Score 3 = poor wound healing with significant gingival edema, erythema, patient discomfort, flap dehiscence, or any suppuration.
Pippi et al. 2015 [11]	20	Oral surgery	1,2 Week	Modified Healing Index (MHI) and pain score	Redness granulation tissue, bleeding, suppuration, swelling, pain on palpation, degree of tissue epithelialization (partial/complete), pain	• Dichotomic score (1/0): 0 presence and 1 point absence with a total score of 7 for better healing, 1–6: equal healing and 0 point worse in comparison to control site healing. • Postoperative pain was evaluated by a 0–10 subjective scale (Pain Score)
Kaner et al. 2017 [20]	30	Periodontal surgeries	D0, D1, D3, D7, and D14	Early healing index (EHI) and dichotomous classification for dehiscence	Complete flap closure, fibrin clot, flap dehiscence	• EHI 1: complete flap closure—no fibrin line in the inter-proximal area • EHI 2: complete flap closure—fine fibrin line in the inter-proximal area • EHI 3: complete flap closure—fibrin clot in the inter-proximal area • EHI 4: incomplete flap closure—partial necrosis of the inter-proximal tissue • EHI 5: incomplete flap closure—complete necrosis of the inter-proximal tissue. Wound closure was assessed dichotomously (soft tissue dehiscence yes/no
Marini et al. 2018 [5]	21	Surgical incisions in periodontal soft tissues	24h	Early healing score (EHS)	Clinical signs of re-epithelialization (CSR), hemostasis (CSH) and inflammation (CSI); Pain perception	Score of 0, 3, or 6 points for the assessment of CSR, whereas scores of 0, 1, or 2 points for CSH and CSI. Higher values indicated better healing (score of 10 for ideal early wound healing was)
Belkhede et al. 2019 [21]	6	Periodontal plastic surgery	1st, 2nd, 3rd, 4th week	Healing index HI	Postoperative discomfort (D), consumption of analgesics during first postoperative week, alteration of sensitivity (AS), change in feeding habits (CFH), complete wound epithelialization (CWE), and healing index	Numerical Rating Scale (NRS) varying from 0–10 score (0 indicating absence and 10 presence) with healing index. CWE was assessed by utilizing hydrogen peroxide
Makki et al. 2021 [22]	88	Oral surgery	2nd & 4th weeks	Landry, Turnbull, and Howley index (LWHI) & Complete Wound Epithelialization (CWE)	Tissue color, response to palpation, suppuration, granulation tissue, and incision margin	5 levels: Very poor/poor/ good/ very good/ excellent

### Wound healing indices

The Healing Index (HI) was introduced by Landry et al. in 1988 [13] and reiterated by Masse in 1993 from the same group to quantify healing after periodontal surgery [13,15]. A score from 1–5 (1 = very poor, 2 = poor, 3 = good, 4 = very good, and 5 = excellent) is given based on evaluating 4 categories: tissue color, response to palpation, granulation tissue, and incision margin.

The Wound Evaluation Scale (WES) evaluated 6 parameters: step-off borders, contour irregularities (puckering), wound margin separation greater than 2 mm, edge inversion (sinking, curling), inflammation (redness, discharge), and overall cosmetic appearance (Quinn & Wells 1998) [16]. Each parameter will be given 0 for yes and 1 for no. A total score of 6 indicates optimal wound healing while lower scores indicate suboptimal wound healing.

The Early Wound Healing Index (EWHI) by Wachtel et al. [17] evaluates guided tissue regeneration (GTR) and focused on wound closure at 1 and 2 weeks in 5 degrees: complete closure (CC), CC+fibrin line, CC+fibrin clot, incomplete closure (IC) with partial tissue necrosis, and IC with total necrosis [17].

A wound healing index by Hagenaars et al. was used for the assessment of periodontal flap surgery on the surgery day and 1, 4, and 8 weeks after surgery [12]. The parameters include swelling of soft tissues (score 0–2), and color of the gingiva (score 0–2), in addition to other standard periodontal parameters, including probing pocket depth (PPD), clinical attachment level (CAL), bleeding index (BI), and plaque index (PI).

A composite index was introduced by Tonetti et al. to evaluate GTR healing up to 12 weeks [18]. Clinical parameters were assessed with a dichotomous scale (presence or absence) of the following: pain, wound closure, wound dehiscence, presence of granulation tissue at wound margin, suppuration, root sensitivity, edema, and hematoma. Patient-centered outcomes were individually evaluated using the visual analog scale (0–100) for the following categories: chewing comfort, esthetic appearance, speaking ability, oral hygiene ability, and overall satisfaction.

The Wound Healing Index (WHI) [19] evaluates periodontal soft tissue wound healing with scores from 1 to 3 [19]. On this scale, a score of 1 indicates uneventful healing with the absence of gingival edema, erythema, suppuration, patient discomfort, and flap dehiscence. A score of 2 indicates uneventful healing with slight gingival edema, erythema, patient discomfort, and flap dehiscence, but no suppuration and a score of 3 corresponds to poor wound healing with significant gingival edema, erythema, suppuration, patient discomfort, and flap dehiscence, or any suppuration [19].

Pippi et al. modified the HI by using the following 7 parameters for extraction socket healing evaluation: gingival color, granulation tissue, tissue epithelialization degree, swelling, bleeding on palpation, pain/tenderness on palpation, and suppuration [11]. Healing was quantified as better, worse, or equal by comparing to the control site. He also evaluated postoperative pain (0–10 scale) based on patients’ perception.

The Early Healing Index (EHI) was used [20] to assess flap closure after periodontal surgeries (GTR and surgical crown lengthening) up to 14 days post-surgery by combining both the Early Healing Index (EHI) [17] and a dichotomous classification (soft tissue dehiscence: yes/no).

The Early Wound Healing Score (EHS) by Marini et al. assesses clinical signs of re-epithelialization, hemostasis, and inflammation 24 hours after periodontal soft tissue surgery [5]. Wounds receive a score of 0, 3, or 6 points for re-epithelialization and 0, 1, or 2 points for both hemostasis and inflammation. Higher values indicate better healing with a score of 10 indicating ideal early wound healing.

The Healing Index (HI) by Belkhede et al. assesses periodontal wound healing up to 4 weeks post-surgery by evaluating postoperative discomfort, complete wound epithelialization, changes in feeding habits, and alteration of sensitivity utilizing the Numerical Rating Scale (NRS), which ranges from 0–10 (0 –absence, 10 –presence) [5,21].

Makki et al. modified the HI by adding complete wound epithelialization (CWE) for evaluating socket healing [22]. A chemical agent, hydrogen peroxide (H2O2) was used for this dichotomous test as either presence of the bubbles to indicate incomplete epithelialization or absence of the bubbles for complete epithelialization.

### Frequencies of the parameters in the included indices

**Fig 2:**
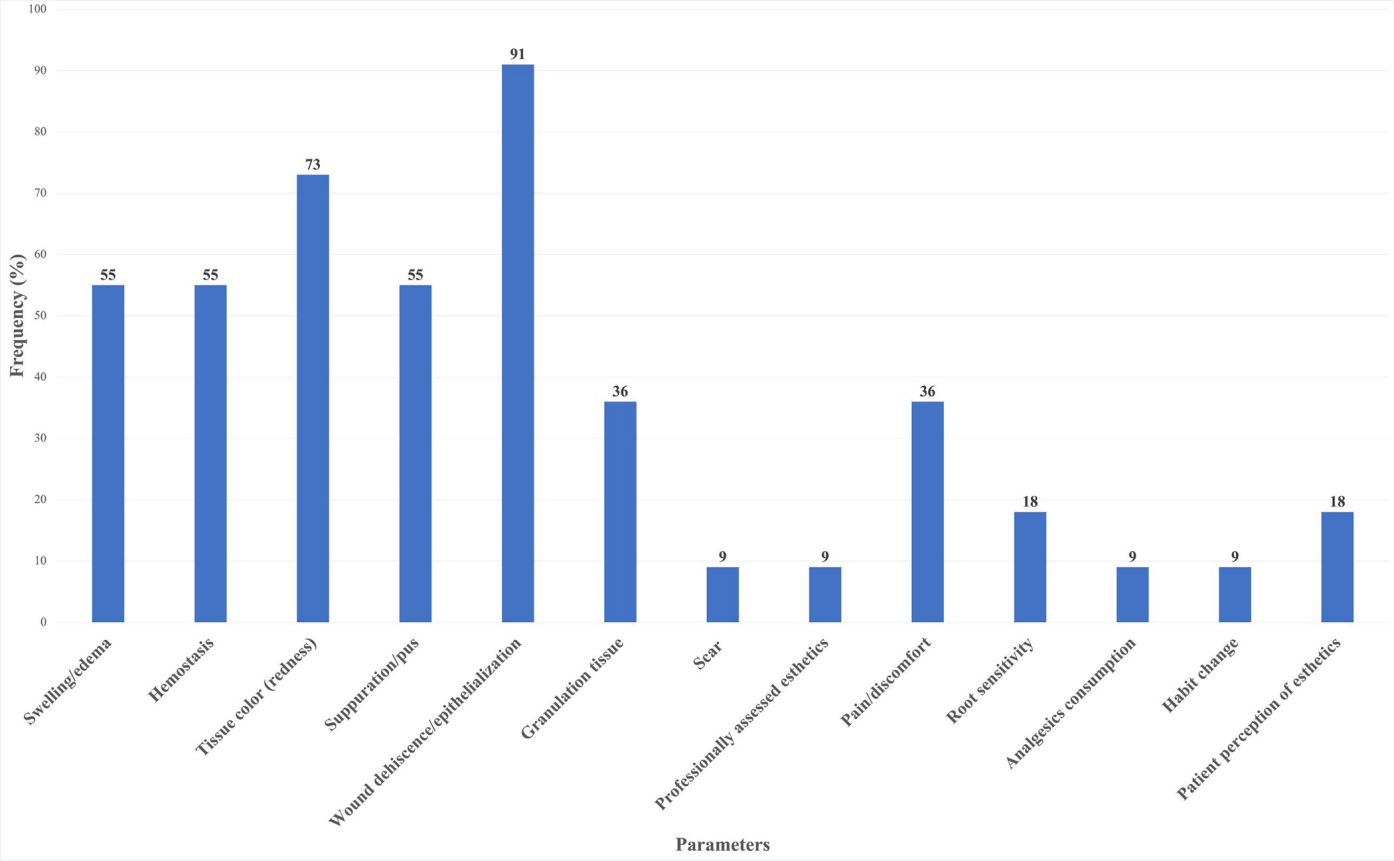
Frequency of clinical signs evaluated on previous indices.

The parameters assessed varied among the included studies, from clinical signs of swelling/edema, hemostasis, tissue color, suppuration/pus, wound dehiscence/epithelialization, granulation tissue, scar formation, professionally assessed esthetics, and patient reports/perceptions. (Fig 2 and Table 2). The frequency of each parameter used in the included indices was calculated as a percentage by dividing the number of times a given parameter was used by the total number of the included papers, which is 11 in our case. Based on this calculation, wound dehiscence/epithelialization is used in all but one included study, followed by tissue color evaluation, used in 8 studies. Swelling/edema, hemostasis, and pus/suppuration were used in 6 studies, equivalent to 55% of the frequency. Granulation tissue and pain/discomfort were assessed in 4 studies, or 36%. Esthetics-related parameters and other patient-centered reports have also been used but much less frequently (Table 2).

**Table 2:** Clinical parameters assessed by the included wound healing indices.

	Inflammation-related			Infection	Tissue-related		Esthetics-related		Patient-centered				
Parameters	Swelling/edema	Hemostasis	Tissue color (redness)	Suppuration/pus	Wound dehiscence/epithelialization	Granulation tissue	Scar	Professional assessed esthetics	Pain/discomfort	Root sensitivity	Analgesics consumption	Habit change	Patient perception of esthetics
Landry et al. 1988 [13]	0	1	1	1	1	1	0	0	0	0	0	0	0
*Quinn et al. 1998 [16]*	1	0	1	1	1	0	1	1	0	0	0	0	0
*Wachtel et al. 2003 [17]*	0	0	0	0	1	0	0	0	0	0	0	0	0
*Hagenaars et al. 2004 [12]*	1	1	1	0	0	0	0	0	0	0	0	0	0
*Tonetti et al. 2004 [18]*	1	1	0	1	1	1	0	0	1	1	0	0	1
*Huang et al. 2005 [19]*	1	0	1	1	1	0	0	0	1	0	0	0	0
*Pippi et al. 2015 [11]*	1	1	1	1	1	1	0	0	1	0	0	0	0
*Kaner et al. 2017 [20]*	0	0	0	0	1	0	0	0	0	0	0	0	0
*Marini et al. 2018 [5]*	1	1	1	0	1	0	0	0	0	0	0	0	0
*Belkhede et al. 2019 [21]*	0	0	1	0	1	0	0	0	1	1	1	1	0
Makki et al. 2021 [22]	0	1	1	1	1	1	0	0	0	0	0	0	1
*Total*	6	6	8	6	10	4	1	1	4	2	1	1	2
*Percentage*	**55**	**55**	**73**	**55**	**91**	36	9	9	36	18	9	9	18

0 = not used; 1 = used.

### Validation of the indices

One out of the abovementioned 11 scales, the WES was validated using data from two cohorts [16]. Data were analyzed retrospectively by two observers comparing the WES with the standard Visual Analog Scale (VAS) on the cosmetic appearance of 91 lacerations and 43 surgical incisions in the face. Intra-observer agreement was calculated using kappa (0.79) and an intraclass correlation coefficient (0.71). As noted in both studies, when an observer gave a good cosmetic score at the first time point the same observer will give a similar score at the later time point. Inter-observer agreement was 0.71, indicating the index is not only reliable but also valid.

## Discussion

Wound monitoring is commonly performed via clinical examination, radiographic assessment, and less commonly via ultrasound and color Doppler imaging [1,7,23–27]. It is a common observation that there is a lack of scale or index to evaluate wound healing for all mucosal disorders [7]. This study reviewed the published indices and dissected them to understand what parameters have been used to evaluate periodontal, implant-related, and oral and maxillofacial surgical wound healing. There is heterogeneity in the parameters used in the indices (Tables 1 and 2) partially due to the variety of procedures studied, including traditional periodontal surgeries, tooth extractions, facial lacerations, periodontal soft tissue reconstruction procedures, and periodontal regenerative procedures. The earliest recall time varied as well, ranging from 24 hours [5] to 2 weeks [13]. The 5 most commonly used parameters are wound dehiscence/epithelialization, tissue color, swelling/edema, hemostasis, suppuration/pus. Primary closure is desirable for periodontal and implant-related regenerative procedures [28]. Studies have shown that early wound exposure is related to inferior bone gain [29]. Wound epithelization starts as early as 2 hours post-surgery to fill the gap between the two margins [30]. Tissue color, hemostasis, and swelling reflect the cellular events in the inflammatory stage. The resolution of this phase and a smooth transition to proliferation/remodeling phases are believed to be critical for optimized healing. Suppuration is apparently an unwanted sign that has to be ruled out. Patient-centered outcomes, such as pain perception are a means to gauge the degree of healing. Prolonged inflammation or underlying infection increases release of pain-related mediators, such as Prostaglandins and Bradykinins that can increase pain intensity and duration [5,11,15,22].

Practically, the timing and frequency of postoperative evaluations are weighed between the benefits and costs of the patients as well as the clinicians. There is no consistency on the timing of assessment through the literature [1,4,5,7,8,11–13,16–21,23–25,27]. The travel expenses and time spent are the obvious costs for such visits. The benefits could be timely and efficient treatment sequences and desirable outcomes. With the increasing application of regenerative procedures, wound evaluations become more important than ever because complications can obliviate the efforts of reconstructing oral hard and soft tissues. Given a relatively high prevalence of soft tissue complications (mean 16.8% [28]) after guided bone regeneration, a frequent checkup regimen may be needed. Early healing assessment may allow for the discovery of complications that would compromise healing and treatment outcomes. This is especially true when non-absorbable membrane is used in patients with thin tissues. When the membrane is exposed and infected, early removal is required to avoid further loss of the grafted bone. Currently, the decision on the timing and frequency of postoperative visits is by and largely arbitrary. These healing indices should be tested for developing an evidence-based and personalized follow-up regimen.

Prior indices assessed patient perceptions (pain/discomfort) [11,18,19,21] and wound esthetic outcome “visually” [16] as being clinically relevant. Nevertheless, scar appearance and clinician-assessed esthetics were only evaluated at the 1-month follow-up [16], while pain/discomfort assessment was followed up for 2 weeks [11], 4 weeks [16], 6 months [19], and 1 year [18]. Due to the increased use of regenerative procedures, improved understanding of wound healing, and the advent of three-dimensional imaging technologies, an assessment of the currently available indices is deemed necessary.

Clinical wound healing indices are not only used to assess the current condition but also serve as a means to inform treatment prognosis. Through the integration of biomechanical, aesthetic, patient-centered, and pathophysiological parameters, it may be possible to gain more useful healing information by monitoring the clinical representations of these critical healing events and evaluating the degree of stability by both visual and functional examinations [1,4,5,7,14,23,31]. The evaluation categories should be distinct without overlapping criteria so the examiners can score a given wound with confidence. In this regard, these indices should be first evaluated for the agreement among the examiners and if they reflect the final outcome. The Wound Evaluation Scale (WES) was validated by comparing to the visual analog scale [16]. This scale primarily concerns about the wound edge appearance. It should be noted that other studies [5,17–21] have modified prior wound healing indices and presented supplemental cases to characterize the assessment of the indices, but these modified indices have not yet been validated. Current available systems are based on subjective wound evaluation. Quantitative analyses of hard- and soft-tissue quality and quantity as well as tissue perfusion could lead to objective wound evaluation.

### Clinical ramifications

Certain oral surgery procedures, particularly periodontal and peri-implant soft and hard tissue regeneration procedures, require meticulous tissue handling during the procedure and wound healing monitoring afterward. Early detection of adverse events triggers more frequent monitoring and intervention if indicated [1,4,5,11,21,22,24–26,31,32]. A careful evaluation of the cardinal signs of inflammation, wound edge status, and patient perception is critical [12,14,18–20,27,28,31,33]. Generally, favorable healing, especially for regenerative purposes is determined by complete wound closure, a smooth healing phase transition, and eventual satisfactory tissue volume gain. The currently available wound healing indices reviewed in this paper revolve around these determinants [12,14,18–20,27,28,31,33]. Moving forward, a simple and validated grading system that incorporates the healing evaluation of a variety of surgical procedures with an emphasis on regenerative and esthetic factors is needed for communication among dental providers. Objective and quantitative indices that can project the final regenerative outcome are needed.

### Research ramifications

Early biological events such as tissue reperfusion, inflammation, and epithelialization play a critical role in wound stability and biomechanical homeostasis [34]. Other biomechanical aspects include flap and suture tension, flap mobility, muscle pull during functional and parafunctional habits, and biomaterial stability can also affect wound stability and biomechanical homeostasis [9,35,36]. Therefore, functional evaluation of wound stability through novel imaging techniques is desirable. Emerging imaging methods including ultrasound and laser speckle imaging can quantify blood flow and tissue elasticity and thus can potentially provide direct measurements of wound healing status [1,26,37–39]. In addition, quantitative data that correlate with wound healing events at the cellular and tissue levels are urgently needed to allow for an objective evaluation of wound healing.

Considering the heterogeneity of the parameters assessed and the timing of the evaluation, a thoroughly wound healing index is deemed necessary. Some key points to include are primary and secondary treatment outcomes, follow-up duration, time of assessment, a criterion for defining success and failure, defect size, clinical measurement [7,11,27,31,40], and the possible use of non-invasive, point-of-care tools to quantify healing i.e., ultrasound, and laser speckle contrast imaging [1,24–26,32,38,39]. This study comprehensively reviewed the currently availvble indices and highlighted the unmet needs that can serve as the framework for future research directions enabling evidence-based wound healing evaluation and outcome assessment [1,5,7,14,22,23,25,27,31].

## Conclusions

Published wound healing indices were systematically reviewed. Considerable heterogeneity existed in the parameters and time points assessed. The indices primarily relied on qualitative wound evaluation and patient perception of wound healing. Quantitative parameters that reflect subclinical healing cascades and biomechanical wound properties, e.g., degree of tissue reperfusion, wound edge characteristics, and biomaterial stability are desirable. These insights could lead to a simple system that objectively predicts the outcome of a procedure and guides monitoring schedules and interventions if indicated.

## Supporting information

S1 Checklist PRISMA 2020 checklist. (DOCX)
